# Housing and Care for Older Women in Australia

**DOI:** 10.3389/fpubh.2021.566960

**Published:** 2021-06-18

**Authors:** Julie E. Byles, Emily M. Princehorn, Peta M. Forder, Md Mijanur Rahman

**Affiliations:** ^1^Centre for Women's Health Research, The University of Newcastle, Callaghan, NSW, Australia; ^2^School of Medicine and Public Health, The University of Newcastle, Callaghan, NSW, Australia; ^3^Centre for Health Service Development, University of Wollongong, Wollongong, NSW, Australia

**Keywords:** housing, healthy ageing, home and community care, residential aged care, longitudinal data

## Abstract

**Background:** Housing is essential for healthy ageing, being a source of shelter, purpose, and identity. As people age, and with diminishing physical and mental capacity, they become increasingly dependent on external supports from others and from their environment. In this paper we look at changes in housing across later life, with a focus on the relationship between housing and women's care needs.

**Methods:** Data from 12,432 women in the 1921–26 cohort of the Australian Longitudinal Study on Women's Health were used to examine the interaction between housing and aged care service use across later life.

**Results:** We found that there were no differences in access to home and community care according to housing type, but women living in an apartment and those in a retirement village/hostel were more likely to have an aged care assessment and had a faster rate of admission to institutional residential aged care than women living in a house. The odds of having an aged care assessment were also higher if women were older at baseline, required help with daily activities, reported a fall, were admitted to hospital in the last 12 months, had been diagnosed or treated for a stroke in the last 3 years, or had multiple comorbidities. On average, women received few services in the 24 months prior to admission to institutional residential aged care, indicating a potential need to improve the reach of these services.

**Discussion:** We find that coincident with changes in functional capacities and abilities, women make changes to their housing, sometimes moving from a house to an apartment, or to a village. For some, increasing needs in later life are associated with the need to move from the community into institutional residential aged care. However, before moving into care, many women will use community services and these may in turn delay the need to leave their homes and move to an institutional setting. We identify a need to increase the use of community services to delay the admission to institutional residential aged care.

## Introduction

As the key foundation for provision of community aged care, “housing” represents an important resource in later life, is a central concern as people age, and is integral to older people's well-being (1). Housing is a source of shelter, purpose and identity, and can be more or less supportive of people's changing needs as they become increasingly susceptible to environmental constraints in later life. As people age into their 80 and 90's, and with diminishing physical and mental capacity, housing becomes more important in protecting the older person from harm, and providing a space where they can receive care and support for their needs.

The housing needs of older people will change with their needs for support, sometimes necessitating dramatic changes in the home environment, either through modifications, moving, or admission to residential aged care in an institutional setting, long term care facility or nursing home (hereafter referred to as residential aged care or RAC). However, research assessing the physical capacities of older people and determining their intentions to modify their homes or move, found that most people were satisfied with their homes and had no plans to move (2). This commitment on the part of the older people was despite the findings that their homes lacked design features to support an older person with greater levels of physical disability (2). Deciding the optimal housing conditions for older people is fraught with difficulty, and often when people do need to move they are least well-equipped to make decisions and to act autonomously.

While remaining in the family home is a key goal for both older people and service providers, the ability to “age in place” is limited not only by health, functional capacity, and available supports, but also by housing type. Recent research highlights the importance of housing as an essential basis for providing care in home and community settings (3–7). Home ownership and arrangements for independent living have also been shown to affect the timing of entry into residential aged care (3, 5, 6, 8–11). However, there is little research on how housing type influences the specific services older people receive. Conceivably, housing type may influence access to services, the type of services that can be provided, the extent to which the older person's needs are met, and the overall goals and outcomes of care. In turn, such services may support an older person to remain in their own home and to continue their participation within their community (3, 12).

In this paper we look at changes in housing and care across later life. We start from a premise that people will want to remain in their own home in the community, and that informal care and formal services can support people to remain in the community with a high level of satisfaction and well-being. We also acknowledge people may alter their housing location and type as they age for various reasons. We further recognise a reality that many people will end their days in RAC, when their needs are too great to remain living in the community even with high levels of support. These housing settings represent a pathway that people can progress along, moving from independent living, to supported care at home, to care in an institutional setting. Along this pathway, different levels of care can support people's functional abilities and ensure a dignified later life (13).

### A Conceptual Model for Housing and Care

**Figure 1:**
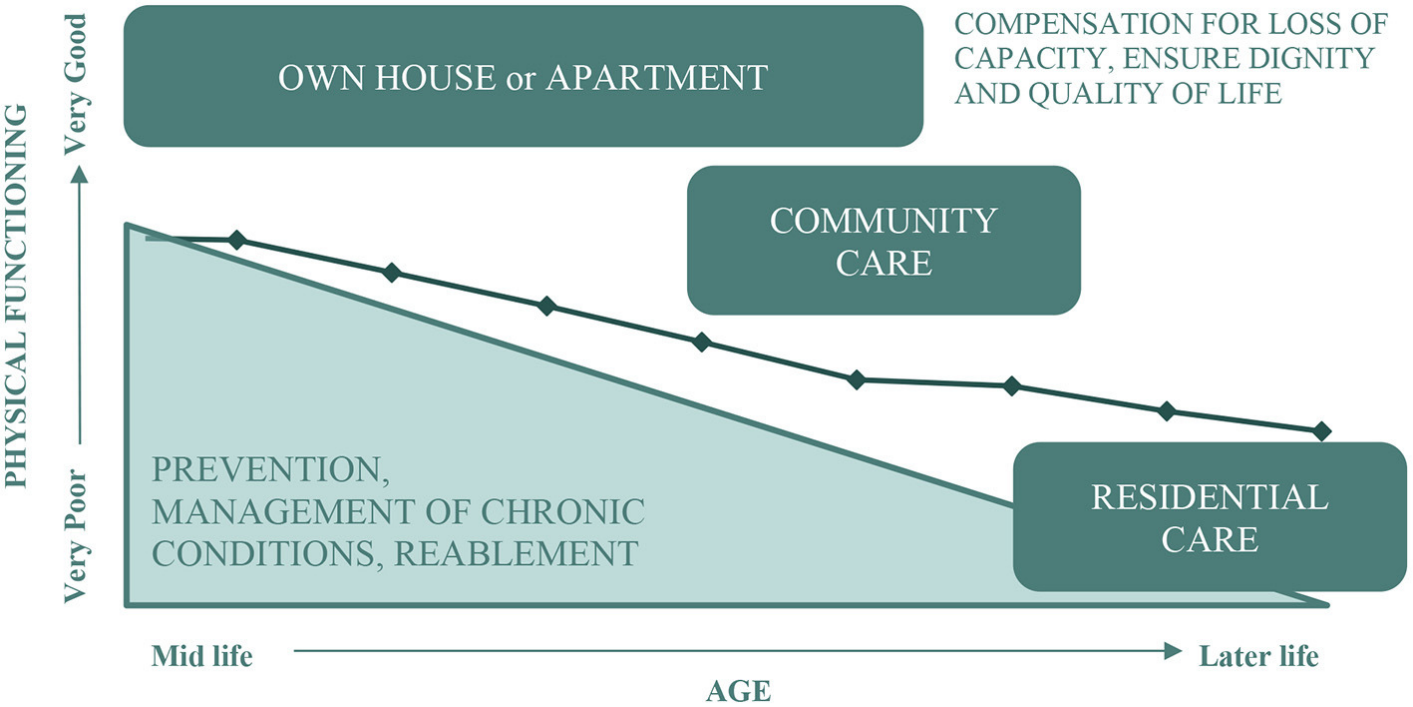
Conceptual model of the association between physical functioning, housing, and the needs for care.

The later period of life can be associated with increasing burden of disease and disability, decline in physical function, decreased capacity for well-being and quality of life, reduced social participation and increased needs for health and social care. However, as shown by Byles (2019), Leigh (2017), and others, there is great heterogeneity of ageing experience, although many people will experience some decline in physical function as they age (14, 15). Figure 1 shows the conceptual relationships between physical functioning, **housing pathways**, and the needs for care as people age (**care pathways**). The line in the figure shows a theoretical age-related decline in physical functioning from very good at younger ages to poorer levels of function in later life. This decline in physical functioning occurs through a dynamic balance between intrinsic capacity and environmental supports. Decline in physical functioning is also associated with decline in cognitive functioning and other capacities, and with increasing disease and comorbidity and need for health care, support from families, and aged services (16). This stage of life can also be a time for transition in housing with some people moving from the family home, to smaller accommodation (downsizing), a village for older people, or residential aged care. The goals for care and support also change according to where people are along this pathway. At earlier ages when people have higher levels of ability, their needs are for supportive environments that enable their participation in family and community life, and encourage prevention of disease and functional decline. These needs remain important as they age and their intrinsic capacities decline, but further needs for support in daily activities to reverse losses and to regain abilities (reablement) begin to have greater priority (see lower left corner in Figure 1). In later life, people need more and more help to compensate for loss of capacity and diminished ability to perform activities of daily living. At the last stages of life, the emphasis of care and support may be to provide for the basics of personal care, and to ensure quality of life and dignity (see upper right corner in Figure 1).

### Housing Pathways

When it comes to housing for older people, Australia's older population faces a clash between personal preferences, cultural values, and social expectations. While most older people in Australia own their own home (1), there is a tension between social and environmental factors that push for moving from the family home into retirement villages and residential aged care facilities, and more personal factors that pull towards remaining in their existing homes. For the most part Australians place cultural value on ageing independently within their own homes, and most older people understandably do not want to move away from their homes and familiar neighbourhoods. These values are supported in Australia with a policy emphasis on ageing-in-place, and by providing care in people's own homes rather than in institutional settings. At the same time, there is a strong social expectation that people will “downsize” into smaller homes and apartments, or move into retirement communities that are designed to meet the specific social and health care needs of older people. This expectation stems from assumptions that older people require less space, or need different types of housing to support their physical needs. The current lack of affordable inner city housing in Australia's capital cities also creates additional pressure to free up valuable housing space. Given this tension, older people find decisions about housing complex and of paramount concern. Moreover, their decisions are not entirely governed by their own preferences but are also influenced by housing markets and social expectations. Residential relocation is also a major life event, and must be considered within the context of an older persons' overall life course.

### Care Pathways in Australia

Most older Australians live in the community, often with their spouse and/or other family members (58%) or alone (25%) (17). While people in their 60 and 70's generally require very little support, as they age into their 80 and 90's they are likely to become frail and experience multiple morbidities and disabilities. These frail older people are particularly dependent on care in order to meet basic daily needs. Mostly these needs are met through informal care provided by family and friends, with support of formal services. Formal aged care in Australia is heavily subsidised by government, with the government responsible for accreditation and accountability of aged care services run by private providers and (for profit and not for profit) organisations. Until recently, these aged care services were provided mainly through Home and Community Care (HACC) in the person's home or in community settings, or through RAC. In more recent years, the HACC program has been replaced with the Commonwealth Home Support Program. This support program provides a range of entry-level aged care services designed to help older people to remain at their home as long as possible by providing practical assistance and social support. Home Care Packages provide more intensive in-home support to those with higher care needs, and seek to avoid admission to RAC.

Home and community care is provided to people living in their own home, either at home or in a community setting. The care can take many forms, including transport, social support, home modifications, home maintenance, meals, allied health (e.g., physiotherapy, occupational therapy, podiatry, dietitian; rehabilitative care to regain function and strength after an illness or injury), and nursing. Profiles of community care service use have been identified by Kendig et al. using data for men and women in the Sax Institute 45 and Up Study (18). This study identified nine different service use groups across these different service types, with most people using few services to meet low level needs across their later life.

RAC is provided in instititutional settings to those older people who are no longer able to be supported in their own home. RAC provides round-the-clock assistance with personal and care needs and many people who have high-levels of dependency, including those with advanced dementia, may be admitted to a RAC facility for full-time care. At any point in time, around 6% of people aged 65 years or over live in RAC (19–21), and <8% of people aged 60 years and over think they will ever need to move into RAC (1). However, the proportion of the population in institutional care increases with age, particularly in the months or years before death. The estimated life-time risk of RAC for people aged 65 and over in Australia is around 40%, with women more likely to be admitted to RAC than men (20, 21).

Respite and Transitional (flexible) care services are also available. Respite allows for a short stay in RAC and allows carers to take a break or to address their own needs. Transitional care is designed as rehabilitation upon discharge from hospital, aiming to enable older people to return home rather than entering permanent RAC.

The assessment of need for aged care services is undertaken by a multidisciplinary team through the Aged Care Assessment Program (ACAP), which provides comprehensive assessment of people with more complex needs. The ACAP assessment includes social, medical, physical, and psychological domains, and clients may be referred to RAC, home care packages, transitional care, and/or respite care. ACAP assessment is essential in order to be eligible for government subsidised RAC. ACAP also has a role in promoting home and community care, in order to prevent or delay admission to RAC (22, 23).

### Pathways of Housing and Care Among Women in Australia

In this paper, we examine the interaction between housing pathways and care pathways for Australian women as they age from their 70's to their 90's, drawing on a wealth of data from the Australian Longitudinal Study on Women's Health (ALSWH). This population study has followed 12,432 women in the 1921–26 birth cohort for 25 years (to date), from when they were aged in their early 70's (in 1996) through to their late 90's, providing information on changes in women's health, housing, and use of health care and aged services (21, 24). The women were randomly selected from the Australian universal health database (Medicare) and with oversampling of women living in rural and remote areas. These women were representative of Australian women in this age group, with slight over-representation of married, Australian born and women with higher level of education. Survey data on a range of demographic, psychological, social and health variables were collected by postal questionnaires every three years from 1996 to 2011 and six-monthly thereafter. In addition to the longitudinal survey data, the study has access to linked data on health and aged care use. These data provide detailed information on the timing and type of services received by the women. The study is also able to ascertain date of death through the National Death Index (NDI) (25). Women generally have longer life expectancy than men and, since they frequently outlive their partners, they often live alone in their later years and have less access to live-in support. We therefore believe that a gendered study looking at the care needs of women is of significance.

A number of papers have already been published reporting on housing and care pathways for women in the 1921–26 cohort of the ALSWH. We summarise these findings here first, before moving to new and additional analyses to examine ACAP assessments of care needs, the use of home and community care, and how these relate to housing type. We are particularly interested in knowing whether women living in a house are more likely to receive ACAP assessments and home care services, compared to women in other housing types.

### Summary and Synthesis of Previous ALSWH Research on Housing and Care

Our first analysis took a longitudinal approach to identify changes in housing type within the context of women's later lives (26). We followed women from the 1921–26 cohort of the ALSWH as they aged from 73–78 to 85–90 years, analysing housing, sociodemographic and health data. The research revealed seven distinct housing patterns that can broadly be categorised as stable, downsize, and transitional. Stable patterns are defined as (1) House (bungalow)—living in a house for most surveys (47.0%); (2) House (bungalow) to the end—living in a house but with earlier death (13.7%); (3) Apartment—living in an apartment (12.8%); and (4) Living in a retirement village/hostel (5.8%). Downsize pattern is defined as (5) Moving from a house to retirement village (6.6%). The remaining patterns are defined in terms of transition from (6) Apartment or retirement village to residential aged care and death (7.8%); and (7) House to residential aged care (6.4%). Few women downsized to an apartment or retirement village, highlighting a disparity between social expectations and the reality for older women.

The vast majority of women remained in a freestanding house giving credence to policy objectives of ageing-in-place. It is also in keeping with our understanding of peoples' attachment to place and the importance of the family home. Stability could also be considered reflective of women's adaptability as well as the ability to modify their environment to suit their changing needs. The person-environment fit also seemed to be important, with women in stable housing patterns tending to be healthier with less need for help with daily tasks. The two transitional patterns reflect the poorer physical health of women moving to RAC with greater need for supportive care.

Hypothesising that some housing types may be more supportive than others, we then examined the association between housing type and the rate of admission to RAC, while also accounting for death as a competing risk. Admission to RAC was strongly associated with age, and with social and demographic characteristics (e.g., education level, marital status/living alone) and health needs (e.g., incontinence, stroke, falls, problems with vision and hearing, levels of physical functioning). Use of RAC was reduced by 13% for women living in rural/remote areas compared to those in major cities. After adjusting for these other factors, participants living in a house had the lowest risk of admission to RAC, while participants living in retirement village/self-care units/hostels had the highest risk. Incidence of admission to RAC over 13 years from their mid 70's to late 80's, was around 27% for women living in a house, 36% for women living in an apartment or townhouse, 44% for women living in a retirement village or self-care unit, and 37% for women living in other types of residences. In contrast, there was little difference in death rates over the 13 years of follow up according to housing type (21).

**Figure 2:**
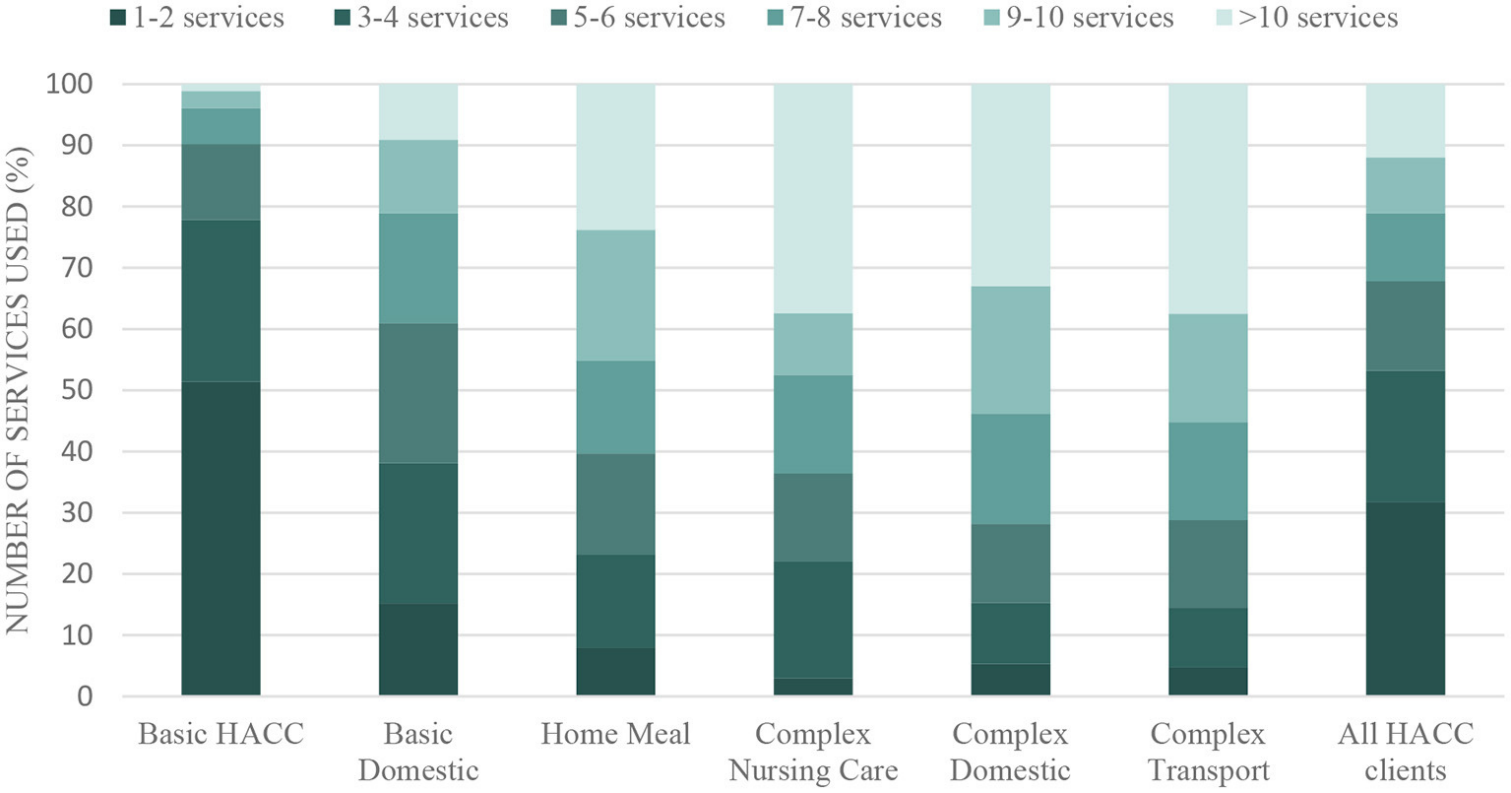
Number of HACC services used by women in different service clusters over the period July 2001 to December 2011. Adapted by permission from Rahman et al. (24).

Our next set of studies examined patterns of use of community care through analysis of the HACC data. This analysis identified six distinct patterns of community care use (provided under HACC). Approximately 54% of the HACC users belonged to a cluster in which women used a minimum volume and number of services and 25% belonged to three complex care use clusters with higher volume and number of services. Significantly higher odds of using HACC were associated with: living in remote or regional areas; being widowed or divorced; difficulty in managing income; not receiving Veteran's Affairs benefits (since Veterans can use an alternative care program), having chronic conditions; lower health related quality of life scores; and poor/fair self-rated health (24). These service use patterns are shown in Figure 2, and indicate a higher use of services such as domestic assistance, transport and meals. These services are considered to be “high volume, low skill.” The services which required more skilled care provision (including personal care, nursing and allied health services) were more concentrated in the complex care groups. Social care and centre-based day care were used by less than one third of the women.

**Figure 3:**
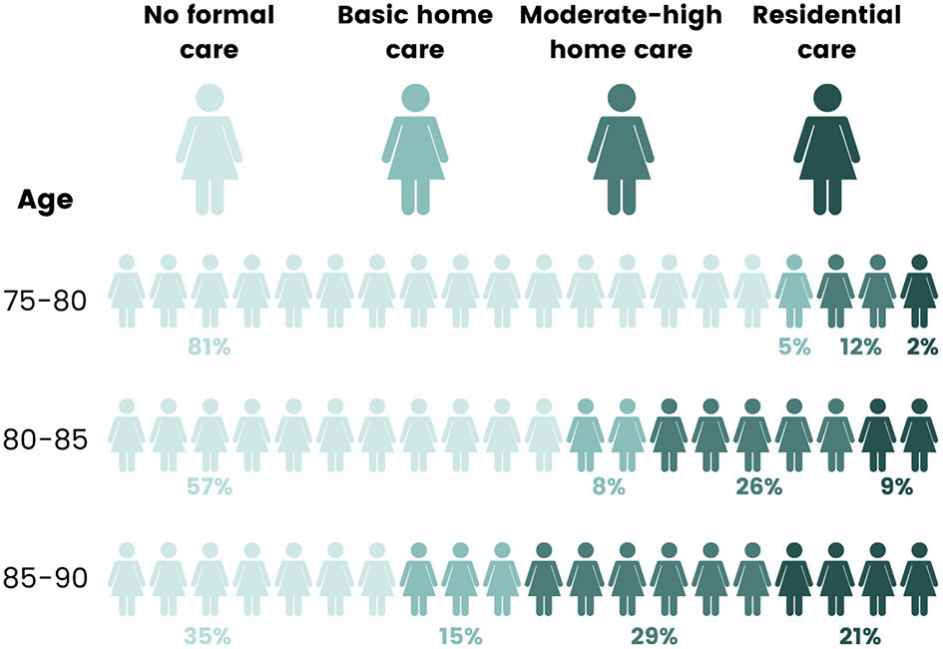
Use of aged care services by women as they aged from 75–80 years (2001) to 85–90 years (2011). Adapted from Rahman et al. (27), with permission from Elsevier.

Using the HACC, RAC and NDI data for each calendar year, we further classified women as either using none of the aged care services, using only limited basic home and community care services, using a larger volume and multiple types of home and community services (moderate to high home care) in RAC, or having died. More than one-third of participants died during the study period, of whom 75% used one or more types of aged care, increasing with proximity to the time of death. Care use among surviving women is illustrated in Figure 3. At age 75–80, most women were not receiving any formal aged care, and only 2% of the women were in RAC. By age 85–90, 21% of surviving women were in RAC, and only 35% were in the community with no formal aged care services (27).

Using latent class analysis, we identified four different pathways through aged care. We found around 40% of women had very low aged care needs, with limited use of home care after age 85. These women had the best physical and mental health and social functioning. Around 25% of women consistently used home care from age 75 onwards, with increased use of RAC in their late 80's and early 90's. A further 10% had high use of RAC from 75 years onwards, and around 25% had earlier mortality and died before their mid 80's, having high use of home care and RAC in the years immediately preceding death. These women had the worst scores for physical function and social function, with more chronic conditions (27).

Women who used home care were more likely to transition to RAC than to remain in home care until the end of life (28). However, we also found that home care use can delay admission to RAC with earlier use of more home services reducing the cumulative incidence rate of admission to RAC. Compared to those who use few basic services, those who used more complex care services had a significantly lower rate of admission to RAC, up to nine years from their first HACC service. After nine years, there was no difference in admission to RAC between the home care groups, indicating that RAC may still be required in later life after a very long period of supported home care (29). Across the cohort, from around age 78, we forecast that, on average, women survive around eight years without using aged care services, five years using home care, and around two and a half years in RAC (28).

Once women enter RAC, they undergo further changes in their health and care needs. These changes can be tracked for the women in the ALSWH using data from the Aged Care Funding Instrument which assesses aged care residents' levels of need across domains of: Activities of Daily Living, behaviours, and complex care needs. Using these data we were able to differentiate women who had been admitted to RAC into five groups. These groups were based on the trajectories of care needs in terms of activities of daily living, behaviours of concern in relation to dementia, and complex health care needs with large variation in the combinations of levels of care needs over time (30). Approximately 28% of residents belonged to the “high dependent–behavioural and complex need” group, which had high care needs in all three domains over time and who tended to be older and have multiple morbidities. Over two-fifths of residents (41%) comprised two trajectory groups (“high dependent–complex need” and “high dependent–behavioural need”), which had medium to high care needs in two domains. Around one-third of residents (31%) were included in two trajectory groups (“less dependent–increasing need” and “less dependent–low need”), which had low or low to medium care needs over time. These latter two groups may represent women who may have been managed in their own home if they had better access to in-home care (28). To explore this possibility, we looked retrospectively at the use of HACC for women who had been admitted to RAC and in accordance with their care need trajectories. The detail on care services provided in Table 1 suggests much room for increased delivery of in-home services prior to admission to RAC. It is possible that RAC admission may have been able to be delayed for many of these women had they received more home support earlier. This is in keeping with our earlier findings on the effectiveness of home and community care in delaying admission to a RAC institution.

**Table 1:** Proportion and median amount of HACC services for older Australian women over the 24 months prior to admission into RAC, according to the five trajectory groups in RAC (*n* = 3,468).

**HACC services**	**Group 1:**	**Group 2:**	**Group 3:**	**Group 4:**	**Group 5:**
	**Less dependent, low need (*n* = 441)**	**High dependent, complex need (*n* = 706)**	**Less dependent, increasing need (*n* = 628)**	**High dependent, behavioural and complex needs (*n* = 954)**	**High dependent, behavioural need (*n* = 739)**
**Domestic assistance**					
% using service	38	40	33	29	29
Median hours (IQR)	40 (13–65)	44 (14–67)	39 (13–64)	29 (10–61)	32 (8–59)
**Meals**					
% using service	28	32	28	23	28
Median number (IQR)	59 (17–201)	72 (17–220)	58 (18–181)	79 (25–218)	98 (22–261)
**Nursing care**					
% using service	27	36	28	36	30
Median hours (IQR)	6 (3–18)	10 (3–26)	9 (3–23)	11 (4–32)	8 (3–22)
**Allied health at home**					
% using service	11	15	12	15	13
Median hours (IQR)	4 (2–7)	3 (1–7)	4 (2–8)	4 (2–8)	4 (1–8)
**Allied health at centre**					
% using service	12	13	11	10	9
Median hours (IQR)	3 (1–6)	4 (1–7)	4 (2–7)	3 (2–7)	3 (1–6)
**Case management**					
% using service	7	9	8	11	11
Median hours (IQR)	3 (1–4)	3 (1–26)	2 (1–3)	4 (2–6)	2 (1–3)
**Care coordination**					
% using service	20	26	22	23	24
Median hours (IQR)	3 (1–6)	3 (1–33)	3 (1–6)	3 (1–8)	3 (1–8)
**Counselling**					
% using service	7	10	11	11	10
Median hours (IQR)	3 (1–4)	2 (1–4)	2 (1–4)	2 (1–6)	2 (1–5)
**Centre based day care**					
% using service	14	15	16	14	17
Median hours (IQR)	117 (24–356)	113 (26–351)	127 (33–327)	192 (38–386)	144 (40–397)
**Home maintenance**					
% using service	18	18	13	14	14
Median hours (IQR)	4 (2–14)	3 (2–10)	4 (2–12)	5 (2–15)	5 (2–13)
**Home modification**					
% using service	5	6	4	8	7
Median AUD$ (IQR)	95 (30–411)	102 (50–284)	64 (31–168)	120 (50–328)	115 (40–306)
**Meals at centre**					
% using service	6	9	9	10	11
Median number (IQR)	22 (6–62)	24 (5–59)	20 (6–43)	22 (5–69)	17 (6–54)
**Nursing care at centre**					
% using service	9	7	6	7	6
Median hours (IQR)	2 (1–4)	2 (1–3)	2 (1–4)	2 (1–4)	1 (1–3)
**Personal care**					
% using service	12	23	16	22	21
Median hours (IQR)	8 (2–18)	11 (3–80)	12 (3–54)	27 (6–101)	16 (4–64)
**Social care**					
% using service	13	21	19	16	19
Median hours (IQR)	9 (3–35)	16 (5–53)	22 (7–59)	17 (5–61)	16 (6–54)
**Transport**					
% using service	23	26	20	20	20
Median number (IQR)	15 (4–78)	18 (4–76)	16 (6–80)	22 (5–82)	18 (4–72)
**Equipment and aids**					
% using service	4	5	4	5	6
Median number (IQR)	3 (2–4)	2 (1–5)	2 (1–7)	2 (1–5)	3 (1–5)

*All services were measured by hours of services except for “Transport” and “Equipment and aids” which were measured by frequence or number of occasions*.

*IQR, Inter-quartile range, for women receiving services; AUD, Australian dollar*.

We have also examined the outcomes of ACAP assessment in terms of uptake of community care or admission to RAC (31). In this analysis, very few women did not access any services after ACAP approval. These individuals had more social resources, and were less likely to live alone. They were also more likely to live in metropolitan areas, where other services may be more accessible. The mean elapsed time for those accessing community care was 159 days, compared to 101 days for those entering RAC. The probability of entering community care was 30% within 100 days, and 53% within 500 days.

For entering RAC, the chances were 20% within 100 days, and 39% within 500 days. These data indicate significant wait times for services, and are consistent with more recent estimates from the Australian Productivity Commision (32). Of those approved for RAC, 47% had mental and behavioural conditions, including dementia, while 62% experienced conditions including amnesia, falls and disorientation. Those accessing community care after the assessment were less likely to have these needs (37 and 57%, respectively). Approval for community-based care was also associated with requiring assistance with communication, health, meals, bodily movement, and self-care. Age was a significant factor for entry into RAC. Living alone increased the rate of access to RAC and decreased rate of access to community care. Having higher education was associated with shorter wait times, and living in an outer regional area was associated with longer wait times for RAC. Entry to RAC was faster if the ACAP assessment took place in a hospital. The results indicate that, while assessed need was the primary driver for access to care, there is also a socioeconomic and geographical gradient (31).

These previous studies illustrate pathways of housing and care, with some evidence that housing type may be associated with more rapid transition into RAC. We also show that earlier and more home and community care may delay admission to a RAC institution, and that the use of these home and community services in the months prior to RAC admission is proportionately low. In the further analyses presented here, we examine whether housing type is associated with greater use of ACAP and/or greater use of home and community care services.

## Measures and Methods

Data were from the 1921–26 cohort of ALSWH linking survey data (Survey 4, 2005, *n* = 7,158 women; Survey 5, 2008, *n* = 5,560; Survey 6, *n* = 4,055; and 6 monthly surveys thereafter), aged care data and National Death Index data for the years 2003 (age 77–82) to 2014 (88–93). Ethical approval for this study was obtained from the Universities of Newcastle and Queensland (Ethics approvals H0760795 and 2004000224). Ethics approval for linkage of ALSWH survey data to aged care and death data was approved by the Australian Department of Health and the Australian Institute of Health and Welfare (AIHW).

### Data on Housing

The housing type variable in this study was obtained at ALSWH surveys using the question “Which of the following best describes your housing situation?” with responses “A house,” “A flat/unit/apartment/villa/townhouse,” “Mobile home/caravan/cabin/houseboat,” “Retirement village/self-care unit,” “Nursing home,” “Hostel,” and “Other.” Missing responses in this variable at a particular time point were filled in using the first valid response from the nearest preceding two surveys. If these were also missing, missing responses were filled using the response at the next survey. Information on admission to RAC was ascertained from administrative data provided by the AIHW (see below). If participants had a record of admission to permanent RAC, housing type at subsequent surveys was updated to “permanent RAC.” Housing type was then categorised into four mutually exclusive groups including “House/Other,” “Apartment/Unit/Flat/Villa,” “Retirement village/self-care unit,” and “RAC/nursing home.”

### Data on Aged Care

Data on aged care were obtained from multiple administrative data bases including the HACC program which provides services to people living in the community, the ACAP which assesses people for their aged care needs, and RAC data on people receiving care in an institutional setting. For those in RAC, we also had data from the Aged Care Funding Instrument which assessed peoples' levels of need across domains of: Activities of Daily Living, behaviours, and complex care needs. These data are linked to ALSWH survey data by the AIHW using a probabilistic linkage algorithm (33). Since the outcomes of interest (home and community care, and ACAP assessment) were ascertained from linked administrative data, there is no concern for loss of follow-up of participants except for attrition by death.

The HACC data were provided for services used/accessed in a calendar quarter (i.e., January-March, April-June, July-September and October-December), with data capturing up to 29 different services options. These service categories were collapsed into 19 main service domains of: counselling (client); counselling (carer); assessment; allied health at home; allied health at centres; centre-based day care; domestic assistance; home maintenance; home modifications; meals (centre); meals (delivered); nursing care at home; nursing care at centre; personal care; respite care; social support; transport; aids; and case management/planning. These data were aggregated for each 12 month period (July-June).

ACAP data included date of assessment, setting of first face to face contact (hospital (acute care)/other inpatient setting/RAC service/other), availability of carer (co-resident carer/non-resident carer/has no carer), current assistance and recommended assistance (yes/no) for self-care, movement, moving around, communication, health, transport, social activity, domestic activity, meals, home entertainment, and other activity. Level of permanent RAC approved (not approved/low/high) was also included. The unit level records were summarised for ACAP assessments for each woman per year (July-June).

### Statistical Analysis

Generalised estimating equations (GEE) were used to model the odds of using HACC services and of having an ACAP assessment according to housing type, adjusted for other demographic and health variables collected on ALSWH surveys. To reflect the women's circumstances at the start of a HACC period, ALSWH survey data from 2005 were aligned with ACAP/HACC usage from July 2005 to June 2008; survey data from 2008 were aligned with ACAP/HACC usage from July 2008 to June 2011; and 2011 survey data were aligned with ACAP/HACC usage from July 2011 to June 2014. A binomial distribution with a logit function and unstructured correlation matrix was used in the GEE models, providing odds ratios (OR) and corresponding 95% confidence intervals. Models initially included each variable adjusted for time period only, and then for each variable including adjustment for all other variables in the model. Women were included in the GEE models up to the period at which they did not return a survey, and so the GEE analysis may be biassed due to non-death attrition. However, previous analyses assess these effects to be small (34).

## Results

As of 2003, there were 10,297 eligible surviving women in the cohort out of the original 12,432 women, but 326 women were already in RAC. Survey 4 (2005, age 82–87) was completed by 7,044 women (eligible for data linkage).

However, 1,998 women were beneficiaries of the Department of Veterans' Affairs and potentially covered by veterans' programs. To avoid underestimation of service use by these women, these women were excluded, leaving 5,046 women for the analysis.

Of the 5,046 eligible women, 2,841 women had ACAP record(s) and HACC record(s). Another 300 women had ACAP record(s) only (no HACC), and 1,331 women had HACC record(s) only (no ACAP). Among the women who had both HACC and ACAP records, 21% had their first HACC service more than five years before their first aged care assessment; 51% had their first HACC service one to five years before their aged care assessment; 20% had their first HACC service in the same year as their first ACAP assessment; 8.0% had their first ACAP assessment one to five years prior to having their first HACC service; and 0.3% had their first aged care assessment more than five years prior to their first HACC assessment. Consistent with our other findings, these data indicate that women were likely to receive HACC well in advance of being assessed by ACAP as potentially needing RAC.

There were 4,158 women (82.4%) who had accessed at least one HACC service between 01 July 2003 and 30 June 2014. Around 55% of these women had used HACC services within the first three years of observation, while 90% of the women had used HACC services within eight years. Table 2 shows the types of HACC services used by these women, based on the first services observed. There were very few differences in the specific services used, according to housing type. In the GEE models, there was also little evidence to suggest that accessing HACC services while living in a unit/apartment (OR = 1.07, *p* = 0.27) or in a retirement village/self care/hostel was different to living in a house (OR = 1.02, *p* = 0.80). The odds of accessing HACC services increased as the women aged, and was higher if women were older at baseline, were not partnered, had difficulty managing on their available income, required help with daily activities, had a fall, or were admitted to hospital in the last 12 months, had been diagnosed or treated for a stroke in the last three years, or had comorbidities (Table 3).

**Table 2:** HACC services for older women in their first calendar quarter of access/use, according to housing status (*N* = 4,158).

**HACC services used (%)**	**House/Other (*n* = 2,916) %**	**Unit/Flat/ Apartment/Villa (*n* = 863) %**	**Retirement village/ Self-care unit (*n* = 379) %**
Aids^a^	2.7	2.3	1.3
Assessment	35.7	36.0	40.3
Allied Health – at home	16.8	18.5	17.6
Allied Health – at centre	3.4	4.9	4.7
Case management/planning^b^	1.0	1.0	0.8
Counselling – client	7.8	8.4	4.2
Counselling – carer	6.8	4.7	5.3
Centre-based day care	5.3	5.4	3.7
Domestic assistance^c^	28.2	26.5	30.0
Home maintenance	13.6	8.9	3.2
Home modifications	5.1	4.6	1.8
Meals – at centre	3.0	4.2	3.7
Meals – delivered to home	9.2	11.5	12.4
Nursing care – at centre	3.0	2.3	0.5
Nursing care – at home	16.5	13.1	17.1
Personal care^d^	4.4	3.2	5.5
Respite care	0.7	0.3	0.5
Social support	5.9	6.5	7.4
Transport	13.5	17.4	20.3

a *Aids and devices for: self-care, support, and mobility, communication, reading, medical care, car modifcations, or other goods/equipment*.

b *Includes case management, case planning and care co-ordination services*.

c *Includes general housekeeping and cleaning activites, as well as “Other food services” not classified elsewhere*.

d *Includes bathing, showering, general hair care, and other toileting care*.

**Table 3:** Effect of housing and other factors on the use of HACC services over time among older Australian women.

	**Adjusted for period only^c^**	**Full model^d^**
	**OR (95% CI)**	**OR (95% CI)**
**Period^a^**		
2005	1.00	1.00
2008	1.77^**^ (1.64, 1.90)	1.79^**^ (1.66, 1.93)
2011	2.64^**^ (2.39, 2.92)	2.53^**^ (2.28, 2.80)
**Housing**		
House/other	1.00	1.00
Apartment/unit/flat/villa/townhouse	1.12 (0.99, 1.26)	1.07 (0.95, 1.21)
Retirement village/self-care unit	1.03 (0.88, 1.20)	1.02 (0.87, 1.19)
**Age at baseline**	1.11^**^ (1.07, 1.15)	1.10^**^ (1.06, 1.14)
**Partnered**		
Partnered (married/defacto)	1.00	1.00
Not partnered	1.29^*^ (1.16, 1.42)	1.23^**^ (1.11, 1.36)
**Ability to manage on income**		
Easy	1.00	1.00
Difficult	1.45^**^ (1.30, 1.62)	1.37^**^ (1.23, 1.54)
**Provide care for others**		
No	1.00	–
Yes	0.94 (0.85, 1.05)	–
**Need help with daily activities**		
No	1.00	1.00
Yes	1.50^**^ (1.32, 1.71)	1.30^**^ (1.13, 1.49)
**Had a fall in last 12 months**		
No	1.00	1.00
Yes	1.22^**^ (1.12, 1.34)	1.15^**^ (1.04, 1.27)
**Stroke in the last 3 years (diagnosed with or treated for)**		
No	1.00	1.00
Yes	1.40^**^ (1.15, 1.70)	1.28 (1.04, 1.57)
**Admitted to hospital in last 12 months**		
No	1.00	1.00
Yes	1.16^**^ (1.07, 1.26)	1.07 (0.98, 1.16)
**Number of comorbid conditions**^b^		
0	1.00	1.00
1–2	1.33^**^ (1.18, 1.50)	1.30^**^ (1.15, 1.46)
3 or more	1.82^**^ (1.59, 2.09)	1.66^**^ (1.44, 1.91)

* *p < 0.05 and*

** *p < 0.01*.

a *Periods of time: 2005- includes period 01 July 2005 to 30 June 2008 (n = 4,252), 2008- includes period 01 July 2008 to 30 June 2011 (n = 3,360), 2011- includes period 01 July 2011 to 30 June 2014 (n = 2,422). The surveys coincided with the start of each period (i.e., 2005, 2008, and 2011)*.

b *Comorbidities include high blood pressure, asthma, bronchitis/emphysema, osteoporosis, cancer, depression, anxiety, angina, heart attack, other heart problems, diabetes, dementia, and osteoarthritis*.

c *Shows association between each variable and HACC service use, adjusting only for time*.

d *Shows association between each variable and HACC service use, adjusting only for time and for all other variables in the model*.

There were 3,141 women (62.2%) who had at least one **ACAP assessment** for aged care services between 01 July 2003 and 30 June 2014. Only 21% of these women had received at least one aged care assessment within the first three years of observation, while 40% of women had an assessment within five years, and nearly 75% of the women had an assessment within eight years. At the time of their first aged care assessment, women who indicated on their previous survey that they were living in a house were more likely to report having a co-resident carer than if they indicated living in an apartment or a retirement village/self-care unit. The first face-to-face setting was similar for women who indicated residing in a house, unit or retirement village (Table 4). At the time of the first aged care assessment, living in a house had the lowest rate of permanent RAC approval (49%), with 52% of women living in apartments having RAC approval, and 59% of women living in retirement village/self-care/hostels having RAC approvals.

**Table 4:** Profile characteristics of the first aged care assessment for older women, according to housing status at the survey preceding the assessment (*N* = 3,137^*^).

	**House/other (*n* = 2,013) %**	**Unit/flat/ apartment/villa (*n* = 693) %**	**Retirement village/self-care unit (*n* = 431) %**
**Location of first contact**			
Hospital (acute care)	16.2	16.2	13.0
Other inpatient setting	5.5	6.1	6.5
RAC service	1.2	1.7	1.9
Other^a^	69.0	68.0	70.5
Not stated/inadequately reported	8.1	8.0	8.1
**Carer availability**			
Co-resident carer	37.5	21.8	17.6
Non-resident carer	40.2	51.7	55.9
Not stated/inadequately described/not applicable	22.3	26.5	26.5
**Current or recommended assistance with:**			
Communications	10.2	8.1	10.9
Domestic activities	90.9	90.3	92.3
Health issues	59.2	55.0	58.5
Home maintenance	72.0	63.5	66.8
Meals	71.5	69.4	75.9
Bodily movement^b^	13.7	12.6	12.3
Moving around^c^	44.5	37.8	44.1
Self-care	48.6	43.6	44.1
Social activities	70.6	70.4	74.5
Transport	81.2	81.0	84.2
Other issues	8.7	9.8	8.6
**Level of permanent RAC approved**			
Not approved	51.4	48.2	41.1
Low	35.2	40.1	48.7
High	13.4	10.8	10.2

* *Four women were excluded due to missing house status at the survey prior to their first ACAP assessment*.

a *Other refers to all other settings such as private residences, outpatient clinics, retirement villages, independent living units, clinic offices, etc*.

b *Bodily movement refers to maintaining or changing position, carrying, moving or manipulating objects, getting in or out of a bed or chair*.

c *Moving around refers to walking and related activities, either around the home or away from home (excludes needing assistance with transport)*.

The results of the longitudinal analyses (Table 5) indicate that the number of ACAP aged care assessments increased over time as the women aged. Having an assessment was more likely if women were living in an apartment (OR = 1.34, 95% CI = 1.18–1.51) or in a retirement village/self-care unit/hostel (OR = 1.47, 95% CI = 1.27–1.69) when compared to women living in a house, even after accouting for other factors. The odds of having an ACAP aged care assessment were higher if women were older at baseline, required help with daily activities, reported a fall, were admitted to hospital in the last 12 months, had been diagnosed or treated for a stroke in the last three years, or had multiple comorbidities.

**Table 5:** Effect of housing and other factors on having an aged care assessment over time among older Australian women.

**Covariates**	**Adjusted with period only^a^**	**Full model*^b^***
	**OR (95% CI)**	**OR (95% CI)**
**Period**^α^		
1: 2005-	1	1
2: 2008-	1.99^**^ (1.82, 2.18)	1.91^**^ (1.73, 2.11)
3: 2011-	3.03^**^ (2.73, 3.36)	2.67^**^ (2.38, 2.99)
**Housing**		
House/other	1	1
Apartment/unit/flat/villa/townhouse	1.31^**^ (1.16, 1.47)	1.34^**^ (1.18, 1.51)
Retirement village/self-care unit	1.46^**^ (1.26, 1.68)	1.47^**^ (1.27, 1.69)
**Age at baseline**	1.15^**^ (1.11, 1.19)	1.13^**^ (1.09, 1.17)
**Partnered**		
Partnered (married/defacto)	1	-
Not partnered	1.05 (0.95, 1.16)	
**Ability to manage on income**		
Easy	1	1
Difficult	1.16^**^ (1.03, 1.30)	1.00 (0.94, 1.06)
**Provide care for others**		
No	1	1
Yes	0.79^**^ (0.71, 0.89)	0.88^*^ (0.78, 0.99)
**Need help with daily activities**		
No	1	1
Yes	3.01^**^ (2.65, 3.43)	2.57^**^ (2.24, 2.94)
**Had a fall in last 12 months**		
No	1	1
Yes	1.55^**^ (1.40, 1.71)	1.37^**^ (1.23, 1.52)
**Stroke in the last 3 years (diagnosed with or treated for)**		
No	1	1
Yes	1.71^**^ (1.42, 2.08)	1.40^**^ (1.15, 1.71)
**Admitted to hospital in last 12 months**		
No	1	1
Yes	1.38^**^ (1.27, 1.51)	1.16^**^ (1.05, 1.27)
**Number of comorbid conditions^β^**		
0	1	1
1–2	1.36^**^ (1.18, 1.56)	1.25^**^ (1.08, 1.45)
3 or more	2.01^**^ (1.73, 2.34)	1.53^**^ (1.30, 1.79)

α *Periods of time: (1) 01 July 2005 to 30 June 2008 (n = 4,252); (2) 01 July 2008 to 30 June 2011 (n = 3,360); (3) 01 July 2011 to 30 June 2014 (n = 2,422). The surveys coincided with the start of each period (i.e., 2005, 2008, and 2011)*.

β *Comorbidities include high blood pressure, asthma, bronchitis/emphysema, osteoporosis, cancer, depression, anxiety, angina, heart attack, other heart problems, diabetes, dementia, and osteoarthritis*.

* *p < 0.05 and*

** *p < 0.01*.

a *Shows association between each variable and HACC service use, adjusting only for time*.

b *Shows association between each variable and HACC service use, adjusting only for time and for all other variables in the model*.

## Discussion

Individuals, families and governments support a shared goal for older people to remain in their own homes in the community. Realising this goal across the majority of later life, for the majority of people, will depend on a constructive balance between optimising the intrinsic capacities of older individuals, minimising disease and disability, providing environmental supports, and providing care. Housing is critical in providing a physically supportive and safe environment, and in offering a place where adequate care can be provided to support frailer individuals in their later years.

Data from the ALSWH highlight the interactions between housing and the use of aged care services over time. We find that coincident with changes in functional capacities and abilities, women make changes to their housing, sometimes moving from a house to an apartment, or to a village. For some, increasing needs in later life are associated with the need to move from the community and into RAC. However, before moving into RAC, many women will use community services and these may in turn delay the need to leave their homes and move to an institutional setting. In this work, we explored whether the housing type influences women's use of community services, assessment for aged care, or transition to RAC.

We found that there were no differences in access to home and community care according to housing type, but women living in an apartment and those in a retirement village/hostel were more likely to have an aged care assessment and had a faster rate of admission to RAC than women living in a house.

Whether this increased need for RAC arises from something particular to housing type, or whether housing type is associatied with other underlying needs is not clear from our studies. We have adjusted for social circumstances, phsyical functioning, and health conditions. However, it may be that living in settings other than a house may be another indicator of social and economic resources, at least within the Australian context. Our study of housing transitions (26) found that most of the women living in apartments had been there for a longer term, with few moving to an apartment in later life, having previously lived in a freestanding home. We have also not accounted for housing tenure, with the possibility that women living in apartments are more likely to be renting or living in social housing. Another Australian study reported that compared to those individuals who owned their own home, individuals living in social housing apartments were most likely to enter RAC (35).

Another possible reason for the protective effect of living in a house, which cannot be assessed in this study, may be that women made modifications to their house, increasing the suitability of the home to their increasing needs. Such changes may not be possible for women living in an apartment. It may also be that remaining in their own neighbourhood rather than moving may also have increased their social connections and reduced their need for care. The finding that people in retirement villages and hostels were more likely to move into RAC may also reflect the increasing needs of these people. From other data, a report by the Australian Institute of Health and Welfare found that, as compared to other housing settings, people living in retirement villages were more likely to be approved for entry into RAC (36). In another study, older people living in group homes or retirement villages tended to use more services than people living in the community (37), again potentially indicating a higher level of need among these people. Whether increasing the services to people in these settings could delay the need for admission to RAC is open to investigation. We and Jorgensen et al. have found that community care can delay the need for RAC. However, our other analyses show that use of services is low and often delayed, and largely independent of housing type except that women living in a house were less likely to have ACAP assessment or be admitted to RAC (38).

Regardless of the setting, older people are highly likely to require some sort of care as they age. Using a life table approach, it has been estimated that more than half of all women in Australia (and a third of men) will be admitted to permanent RAC at some time in their life (39), and a greater proportion will need some form of community care. Meinow's study in Sweden found that almost all older people required aged care in the last two years prior to death, either in their own home or in an institutional setting (40). In the same study, older age at death was associated with increased use of aged care services, even when adjusting for closeness to death. Likewise, modelling of longitudinal data from Japan estimated that at age 78, around 90% of women had no long term care, but the probability of surviving women transitioning from no care to some care within three years was 15% for community care, and 5% for institutional care. After six years, 31% of surviving women would be receiving community care and 7% would be receiving care in an institution (41). In our own analysis, most women used some form of aged care in their later life, with only 28% of women using no formal aged care services, most women using home and community care, and some transitioning into RAC. Most of those who entered RAC had already used some form of community care (28). These findings are also similar to national data reported by the Australian Institute of Health and Welfare (AIHW), showing that over two-thirds of residents in RAC had first used home and community care (42).

For some women, RAC may be the only way to adequately meet their needs. However, our data and data from other studies support that home and community care may have an important role in supporting people to stay at home and to delay or avoid admission to an institution (29, 43).

On the other hand, some women who need to enter RAC in order to support their high levels of need may be less likely to do so if they have inadequate financial assets (30). While RAC is subsidised by the Australian government, people with equity in their own home can draw down on this asset to pay for premium care with greater choice, comfort levels and extra services. Home ownership therefore may operate as a form of long term care insurance within the Australian context. A recent report published by the Centre of Excellence in Population Ageing Research considers this role of the family home in providing financial security in older age. Thus, the home can be considered not only as the place where care can be provided, but also as the means by which care may be afforded (44). The decline in home ownership in Australia may have important implications for the financing and affordability of RAC.

For women who have moved into RAC, the care facility should be seen not as an institution, but as their home. Given that most women who enter RAC will remain in that facility for at least two and a half years in our study (28) or longer (42), the RAC home is likely to become domicile, community and social network. The social and physical environment of the RAC setting is therefore another area of concern in considering the nexus between housing and care. Homelike environments can be promoted through design, the designation of private and shared places and through the ability to personalise their space. Safety, security and sense of autonomy also remain important for people in these settings (45).

Given the need for care as people age, and the context of population ageing which will see increasing demands for care, detailed information on housing transitions and use of services is vital for planning of aged and social care services, and to meet the needs of older people. Despite a preference for older people to live at home, there are few evaluations of the benefits of community care vs. RAC and of which home care services are the most beneficial (46). There is some concern that home care is fostering social isolation and that some older people would be better off in a RAC setting. There is a further issue that older people find it very difficult to navigate the aged care system, particularly in deciding what services they need and the potential benefits vs. perceived costs. This issue of identifying and selecting appropriate services may be particularly true for services that have a preventive or reablement approach that enables the person to participate in daily activities, as compared to services such as meals, shopping and cleaning that substitute for what the older person can no longer do. Older people could be better supported to make choices for their aged care, and their accommodation choices, if they had information about the benefits of different services, including those that will enable their continued independence and enable them to stay in their own home. Improvements to community care include greater provision for reablement needs to be assessed and reablement approaches to be embeded in the services provided (47). There is also need for better integration between health care and community supports (48), and for a stronger evidence-base for reablement approaches (49, 50). There is also a recognised need for better palliative and end-of-life care (46, 51).

In Australia, there has been a Royal Commission into Aged Care Quality and Safety which has identified the challenges faced by older people deemed eligible for a Home Care Package. These challenges are a result of older people, firstly, having to wait in the national prioritisation queue before a package of services is “assigned,” and then, having to find a service provider to deliver their care (52).

The report acknowledged that this process can “*take a very long time*,” especially for those who have higher care and support needs, with many people dying while waiting for a Home Care Package and others prematurely moving into RAC. There is also a widely recognised need to improve RAC for older people. The report from the Royal Commission, poignantly titled “Care, Dignity and Respect,” acknowledged that people often enter RAC with great trepidation with fear of loss of individuality, autonomy and control over their own lives (52). This is particularly concerning, given ALSWH data indicates that around 40% of women will be admitted to permanent RAC at some point in their last years (around 80% for those with dementia), with two and a half years average length of stay (26), and rapidly increasing needs over the course of their aged care stay (25).

### A Comment on Older Men

Research on ageing has tended to focus on women, who have a longer life expectancy than men, and make up the greatest proportions of people at oldest ages. However, men's life expectancy is increasing, and a growing proportion of older people are men (53). Compared to women, men are more likely to be partnered, living at home in a private dwelling (17), and to have someone to care for them (54). However, as men's life expectancy increases, a larger number of men are living to very old age, and increasing proportions live alone (53). These older men who live alone may have little in the way of informal support (55, 56), and may have greater mental and physical health needs and poorer health risk behaviours, compared to men who live with a partner (57), or to women. There is however little information on the health and care needs of older men. One study examined data from the Sax Institute 45 and Up Study to compare the mental health and physical functioning of community-dwelling men aged 70 years or over who live alone, and those who live with their partner or spouse. Among this large population of community-dwelling older men, those who did not have a spouse or partner had more adverse health risks (such as smoking, drinking and low BMI), and up to age 85, had poorer physical health-related quality of life and were more likely to have poor mental health compared to men with a partner (58).

As more men reach older ages, the number of older men requiring aged care is also increasing. However, in 2019, only one-third of people using aged care services in Australia were men (19). In their recent Australian study, Khadka and colleagues compared incidence rates of aged care service utilisation between 2008–09 and 2015–16. While the proportion of men utilising aged care services had increased slightly from 2008 to 2009 levels, they still only made up 39.7% of admissions to RAC in 2015–2016 (59). In comparing men and women's use of community services, Kendig et al. found that men were less likely to use transport services and domestic assistance, but more likely to use nursing services (18). Unpartnered men and women were more likely to use nursing services. Generally, men were less likely to use home and community care than women were, and people without a partner used more services than people with a partner (18).

The reasons that men do not use aged care in as great a proportion as women are likely to be multiple. In addition to their shorter longevity and greater likelihood of being partnered, they also tend to have lower levels of disability than similarly aged women and are less likely to be frail (60). It has also been suggested that masculine attitudes to help seeking may operate as a barrier to men accessing care. In one study undertaken in Australia, men saw aged care services as feminised and not “male friendly” (61). These gender issues are an important consideration in ensuring services are appropriate for older people, and not simply available.

## Concluding Comments

Based on our findings we would argue that much needs to be done to increase access to more community services earlier, when women are living in their own homes, and to increase the opportunities for prevention and reablement within the aged care system. We also strongly believe that the system needs to encourage people to plan ahead for their ageing and aged care needs, with a focus on their goals, their identity and their right to make informed choices about their care and where they choose to live. We further assert that women should not have to leave their homes which have meaning to them to receive the care that they need.

We see aged care as “the long tail” of the prevention pathway with a focus on long term ability to engage meaningfully in life, long term dignity, and long-term autonomy. Even very old people can maintain their capacities, regain strengths and abilities, and can prevent future loss and decline with the appropriate support and social environments. Even when people's intrinsic capacities are greatly diminished and compromised, there is much that can be done to support their well-being. This balance between housing and aged care, as supporting people even when their intrinsic capacity is diminishing is in keeping with the World Health Organizations' goal of healthy ageing, helping older people to maintain their ability to “do the things they have reason to value.”

## Data Availability Statement

Information on applying for ALSWH data is available from https://www.alswh.org.au/for-datausers/applying-for-data/. Data Access enquiries can be directed to this e-mail: alswh@uq.edu.au.

## Ethics Statement

The studies involving human participants were reviewed and approved by The University of Newcastle Human Research Ethics Committee. The patients/participants provided their written informed consent to participate in this study.

## Author Contributions

JB conceptualised and designed the studies reported here and wrote the first draught of the manuscript. PF and MR led and performed the analysis of the data and drafted the methods and results. EP wrote sections of the manuscript. All authors contributed to manuscript revision, read, and approved the submitted version.

## Conflict of Interest

The authors declare that the research was conducted in the absence of any commercial or financial relationships that could be construed as a potential conflict of interest.
